# Expression of sphingosine kinase 1 and sphingosine 1-phosphate receptor 3 in malaria-associated acute lung injury/acute respiratory distress syndrome in a mouse model

**DOI:** 10.1371/journal.pone.0222098

**Published:** 2019-09-04

**Authors:** Chuchard Punsawad, Parnpen Viriyavejakul

**Affiliations:** 1 School of Medicine, Walailak University, Nakhon Si Thammarat, Thailand; 2 Tropical Medicine Research Unit, Research Institute for Health Sciences, Walailak University, Nakhon Si Thammarat, Thailand; 3 Department of Tropical Pathology, Faculty of Tropical Medicine, Mahidol University, Bangkok, Thailand; Flinders University of South Australia, AUSTRALIA

## Abstract

This study aimed to investigate the expression of sphingosine kinase 1 (SphK-1) and sphingosine 1-phosphate receptor 3 (S1PR-3) in a mouse model of malaria-associated acute lung injury/acute respiratory distress syndrome (ALI/ARDS). DBA/2 mice were infected with *Plasmodium berghei* ANKA to generate an experimental model of malaria-associated ALI/ARDS. The infected mice were divided into 2 groups based on the histopathological study of lung tissues: those with and those without ALI/ARDS. The expression of the SphK-1 and S1PR-3 proteins in the lung tissues was investigated using immunohistochemical staining and Western blot analysis. In addition, the S1P level was quantified in plasma and lung tissues using an enzyme-linked immunosorbent assay (ELISA). The results demonstrated that the cellular expression of the SphK-1 and S1PR-3 proteins was significantly upregulated in endothelial cells, alveolar epithelial cells and alveolar macrophages in the lung tissues of malaria-infected mice with ALI/ARDS compared with those in the control groups. The increased expression of the SphK-1 and S1PR-3 proteins was confirmed using Western blot analysis. The concentration of S1P in plasma and lung tissues was significantly decreased in malaria-infected mice with ALI/ARDS compared with non-ALI/ARDS and control mice. Furthermore, increased expression of the SphK-1 and S1PR-3 proteins significantly correlated with lung injury scores and S1P concentrations in malaria-infected mice with ALI/ARDS. These findings highlight increased expression of SphK-1 and S1PR-3 in the lung tissues of malaria-infected mice with ALI/ARDS.

## Introduction

According to the World Health Organization malaria report, there were 216 million malaria cases worldwide in 2016, leading to approximately 445,000 deaths [1]. Infection with plasmodium parasites may result in severe malaria with pulmonary complications, such as acute lung injury (ALI) and acute respiratory distress syndrome (ARDS), which often develop several days after the initiation of antimalarial treatment, causing up to an 80% increase in morbidity and mortality [2]. Several previous studies have reported that DBA/2 mice infected with *Plasmodium berghei* ANKA can develop ALI/ARDS, characterized by increased vascular endothelial growth factor (VEGF) and lung permeability, resulting in the development of pulmonary oedema and alveolar haemorrhage [3, 4]. The pathological alterations of lungs with ALI/ARDS as observed by haematoxylin and eosin staining include alveolar septal thickness, capillary congestion, and several cellular infiltrations in the pulmonary alveolar space, including neutrophils, macrophages, infected red blood cells, and non-infected red blood cells as well as hyaline membrane formation [2, 5, 6]. Ultrastructure studies of the lung in a murine model of malaria-associated ALI/ARDS revealed infected red blood cells binding to the endothelium, endothelial swelling with cytoplasmic extension, and thickness of the endothelial basement membrane and alveolar septa [7]. Further study regarding the pathogenesis of malaria-associated ALI/ARDS demonstrated that haemozoin, the waste product of the *Plasmodium* protozoan, was associated with pulmonary complications, as evidenced by the increased levels of VEGF, proinflammatory chemokines, cytokines, and other inflammatory mediators in *Plasmodium falciparum*-derived haemozoin-injected mice [2, 3, 5, 6, 8]. However, the pathogenesis of pulmonary complications in malaria is still poorly understood. Further studies are needed to provide important insights into the mechanisms involved in the pathogenesis of ALI/ARDS during malaria infection.

Sphingosine 1-phosphate (S1P) is a bioactive sphingolipid generated by sphingomyelin metabolism that acts almost ubiquitously, influencing many key biological parameters, including cell proliferation, differentiation, motility, survival, and the regulation of immune function. [9–12]. S1P is generated by the phosphorylation of sphingosine catalysed by sphingosine kinases (SphKs), namely, SphK-1 and SphK-2 [13, 14]. Human SphK-1 consists of three isoforms, isoform a (42.5 kDa), isoform b (51 kDa) and isoform c (43.9 kDa) [15, 16], for which the highest expression levels are found in the lungs, spleen, and liver [13, 14, 17]. Human SphK-2 is approximately 65.2 kDa [18] and expressed as five isoforms: isoform 1 (a), isoform 2 (b), isoform 3 (a), isoform 4 (c) and isoform 5 (d) [15]. Human SphK-2 is most abundantly expressed in the kidney, liver, and brain [18]. S1P can act in an autocrine or paracrine manner that is mediated by a family of five specific cell surface G-protein-coupled receptors, namely, S1PR1-5 [9, 10]. S1P and its receptors have been proposed to play a role in the vascular and immune systems [19]. S1PR3 is mainly expressed on vascular endothelial and smooth muscle cells [19] and may be involved in vascular integrity disruption in ALI [11, 20, 21]. Binding of S1P to S1PR3 also induces the release of Weibel-Palade bodies, organelles in endothelial cells that store various proteins involved in inflammation [22]. Regarding previous reports focused on S1P in malaria, reduced levels of S1P have been previously demonstrated in Ugandan children with cerebral malaria [23] and in complicated *P*. *falciparum* malaria patients on admission and day 7 post-treatment [24]. Sun and colleagues demonstrated increased circulating plasma concentrations of S1PR-3 in mice and humans with ALI, representing a novel ALI biomarker linked to disease severity and outcome [21]. Although increasing evidence suggests the involvement of S1P in malaria, very little is known regarding the role of SphK-1 and S1PR-3 in malaria-associated ALI/ARDS. We hypothesized that S1P might mediate ALI/ARDS via a signalling pathway involving the conversion of sphingosine to S1P by SphK-1; then, binding of S1P to S1PR-3 leads to receptor activation that promotes the development of malaria-associated ALI/ARDS. To understand the roles of SphK-1 and S1PR-3 in lung tissue, this work analysed the expression and distribution of SphK-1 and S1PR-3 and their relationship in a mouse model of malaria-associated ALI/ARDS. We employed DBA/2 mice infected with *P*. *berghei* ANKA as a model of malaria-associated ALI/ARDS mimicking human ALI/ARDS in severe malaria [3].

## Materials and methods

### Preparation of animals and experimental protocols

Six- to ten-week-old DBA/2 male mice purchased from BioLASCO Co., Ltd. (Taipei, Taiwan) were used in this study. The mice were housed five animals per cage in standard polycarbonate cages with wood shaving bedding, with free access to food and fresh water, temperatures ranging from 22 to 24 °C, and a standard 12-h light/dark cycle, until experimental use. The mice were intraperitoneally (i.p.) injected with 1 × 10^6^ *P*. *berghei* ANKA-infected red blood cells as previously described [3, 25]. The malaria parasites were obtained through BEI Resources, NIAID, NIH: *P*. *berghei*, Strain ANKA, MRA-311, contributed by Thomas F. McCutchan. Control mice (n = 10) received saline only (200 μl, i.p.). All mice were monitored daily for health, including general appearance, interaction with cage mates and hands-on physical examination for hydration status and physical abnormalities. Parasitaemia levels were monitored daily using Giemsa-stained peripheral blood smears. All mice were sacrificed on the 13^th^ day after infection to ensure that ALI/ARDS had already occurred, and histopathological examination of the lung was ultimately performed by a pathologist for group classification as non-ALI/ARDS and ALI/ARDS. Anaesthesia was administered by the intraperitoneal injection of 0.8% pentobarbital sodium (60 mg/kg). Once anaesthetized, blood was collected by cardiac puncture for the measurement of S1P, and lung tissues were harvested immediately for histopathological, immunohistochemistry and Western blot analyses. In addition, protein was extracted from lung tissues and stored at -80 °C until use. Based on the histopathological study of lung tissue, 10 out of 20 *P*. *berghei*-infected mice developed ALI/ARDS. The infected mice were divided into 2 groups, those with (n = 10) and without (n = 10) ALI/ARDS. This study protocol was approved by the Animal Ethics Committee, Walailak University (Protocol no. 001/2560).

### Histopathological examination of lung injury

The lung tissues were fixed in 10% formalin for 24–48 h and embedded in paraffin. Paraffin blocks of lung tissue were sectioned at a thickness of 5 μm and stained with haematoxylin and eosin. A histopathological scoring method was used to quantify changes in the lungs under a light microscope at high magnification (400×) by two independent observers (CP & PV) who were blinded to the experimental groups. The degree of microscopic injury was assessed using 4 categories of histologic changes, including alveolar oedema, alveolar haemorrhage, septal thickening and leukocyte infiltrate. The severity of these categories was scored by a previously described five-point system [26] with minor modifications as follows: 0, no injury; 1, injury to 25% of the high power field; 2, injury to 50% of the high power field; 3, injury to 75% of the high power field; and 4, diffuse injury to > 75% of the high power field. Finally, to quantify the overall histopathological changes, a lung injury score was calculated by adding the four category scores, for a total of 0–16. A score of 0 indicates no injury, while a score of 16 indicates maximum severity.

### Expression of SphK-1 and S1PR-3 by immunohistochemistry staining

To investigate the cellular expression of the SphK-1 and S1PR-3 proteins, lung sections were deparaffinized in xylene and rehydrated in a series of ethanol dilutions. Sections were incubated for 10 min in 3% hydrogen peroxide to quench endogenous peroxidase. To improve antigen retrieval, sections were incubated in antigen unmasking solution (Vector Laboratories Inc., USA) for 30 min in a microwave. After washing in Tris-buffered saline (TBS), sections were blocked for 30 min with normal goat serum. Sections were then incubated for 1 h at room temperature with rabbit polyclonal against SphK-1 (1:200; Ab71700, Abcam, UK) or S1PR-3 (1:200; sc-30024, Santa Cruz Biotechnology, Inc., USA) as a primary antibody, followed by a 30-min incubation with diluted biotinylated secondary antibody and then a 30-min incubation with VECTASTAIN ABC Reagent (Vector Laboratories Inc., USA). Then, all sections were incubated in 3,3'-diaminobenzidine reagent (Vector Laboratories Inc., USA) for brown chromogen staining, counterstained with haematoxylin, rehydrated, and mounted for analysis using a light microscope. Known positive controls (mouse liver tissue) for SphK-1 and S1PR-3 were used to confirm the specificity of primary antibodies and validate the immunohistochemistry staining techniques. Laboratory methods for the negative controls were performed in the same manner but without primary antibodies. To evaluate the expression of SphK-1 and S1PR-3, each immunostained section was randomly counted in 10 microscopic fields at high magnification (400×) for positive cells and intensity of target proteins. The expression levels of SphK-1 and S1PR-3 were separately examined in endothelial cells, alveolar epithelial cells and alveolar macrophages based on morphology and location of each cell type. Microscopically, endothelial cells are very flat cells with central nuclei, which are located on the inside of the blood vessels. Two types of alveolar epithelial cells, namely type I and II alveolar epithelial cells, were observed in the alveolar wall. Type I cells are thin and flat, whereas type II alveolar epithelial cells are cuboidal. Alveolar macrophages are located on the internal surfaces of alveolar septum or within the sinusoidal space. They are pleomorphic in shape with a dark or black-brown accumulation in the cytoplasm. For semi-quantitative analysis, the percentage of positively stained cells for each protein marker was calculated by dividing the number of positive cells by the total cell count and multiplying this number by 100. The intensity of staining with both antibodies was subjectively scored as follows: 0, no staining; 1, weakly positive; 2, moderately positive; and 3, strongly positive. Finally, the total score was calculated by multiplying the percentage of positive cells (%) by the staining intensity (I), according to a previous study [27].

## Preparation of lung homogenates

Frozen mouse lung tissues isolated from control mice, malaria-infected mice with non-ALI/ARDS and malaria-infected mice with ALI/ARDS (5 mice per group) were homogenized in cell lysis buffer (Cell Signaling Technology, USA) containing protease inhibitor (Roche, Switzerland). After homogenization, samples were kept for 1 h on ice following a 10-min centrifugation at 1600 rpm at 4 °C to collect the supernatant (protein extract). The protein concentration of each specimen was measured based on the Bradford method utilizing the Bio-Rad Protein Assay Kit (Bio-Rad Laboratories, USA) with bovine serum albumin (BSA) as the standard.

## Detection of SphK-1 and S1PR-3 by Western blot analysis

Proteins from 20 mg of each of the extracts were separated by 12% SDS-polyacrylamide gel electrophoresis (PAGE) gel (Bio-Rad Laboratories, USA) at room temperature and then transferred onto a polyvinylidene fluoride (PVDF) membrane (Bio-Rad Laboratories, USA) at 4 °C. After blocking in 10% TBS-0.01% Tween 20 (TBS-T) diluted with non-fat milk (Amresco, USA) for 2 h at room temperature, the membranes were then incubated overnight at 4 °C with primary antibodies: rabbit polyclonal antibody to SphK-1 (1:300; Ab71700, Abcam, UK) or S1PR-3 (1:300; sc-30024, Santa Cruz Biotechnology, Inc., USA), with β-actin (1:1000, Ab8227, Abcam, UK) as the positive control. After washing the membrane with TBS-T solution, HRP-conjugated goat anti-rabbit secondary antibody (1:5000; ab6721, Abcam, UK) was added, and the membranes were then incubated for 1 h at room temperature. After washing, protein expression was visualized using an enhanced chemiluminescence (ECL Plus) assay kit (Amersham Biosciences, UK) according to the manufacturer’s instructions. Expression of the SphK-1 and S1PR-3 proteins of the SphK-1 and S1PR-3 bands in the immunoblot relative to the housekeeping protein β-actin was calculated by densitometry using ImageJ software.

### Measurement of S1P concentrations by ELISA

The concentration of S1P was determined in plasma and lung tissue of the control and experimental groups by using a standard sandwich ELISA kit (Echelon Biosciences, Inc., USA) according to the manufacturer’s instructions with modification, as previously described [24]. Each sample was analysed in duplicate. The results are presented as the mean ± SEM.

### Statistical analysis

Statistical analysis was performed using IBM SPSS scientific statistics version 23.0 software (SPSS, IL, USA). All quantitative data are presented as the mean ± standard error of the mean (SEM). The normality of distribution was tested using the Kolmogorov-Smirnov test. Differences between groups were analysed by a non-parametric Mann-Whitney U test. The correlation between the associations between SphK-1, S1P and S1PR-3 expression was computed by Spearman’s rank correlation to estimate the direction and strength coefficient. A *p* value < 0.05 was considered significant.

## Results

### Histopathological assessment of lung injury

**Fig 1:**
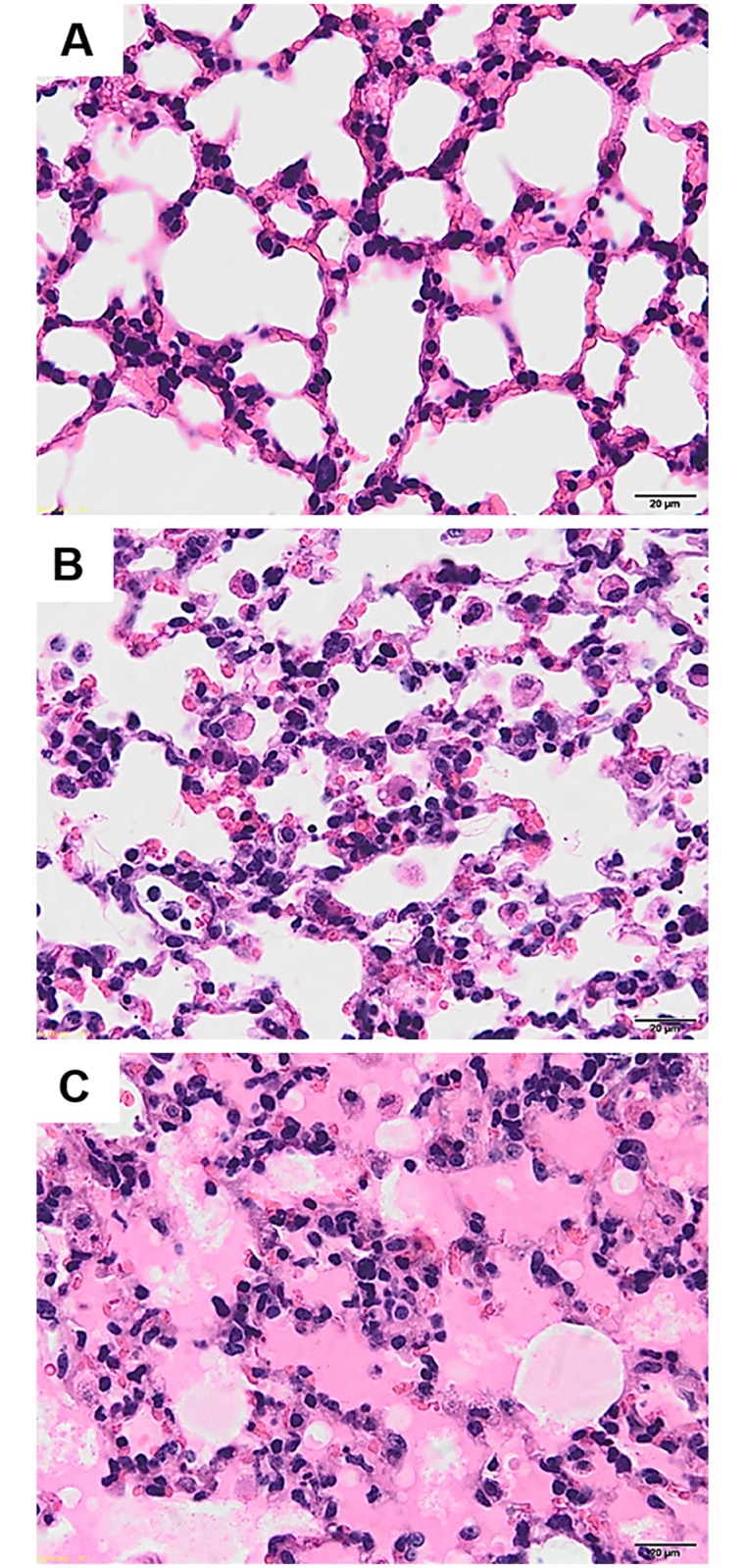
H&E-stained lung tissues from malaria-infected mice with ALI/ARDS showed more severe histopathological changes than those from control mice and malaria-infected mice without ALI/ARDS. (A) Normal lung tissues in the control mice (n = 10). (B) The lung tissues of malaria-infected mice without ALI/ARDS (n = 10) showed slight septal thickening and haemorrhage with few leukocyte infiltrates. (C) The lung tissues of malaria-infected mice with ALI/ARDS (n = 10) showed alveolar oedema, alveolar haemorrhage and thickened septa filled with congested capillaries, infected red blood cells and leukocytes. All images are 400× magnification. Bar = 20 μm.

Histopathological evaluations indicated that the lungs from malaria-infected mice with ALI/ARDS differed from those from the control mice (Fig 1A) and those from malaria-infected mice with non-ALI/ARDS (Fig 1B). The ALI/ARDS lungs showed alveolar oedema, alveolar haemorrhage and thickened septa filled with congested capillaries, infected red blood cells and leukocytes (Fig 1C). Table 1 presents the semi-quantitative analysis of the histopathological changes in the lung tissues according to our scoring system. The degree of microscopic injury in the group of malaria-infected mice with ALI/ARDS was significantly higher than that in malaria-infected mice with non-ALI/ARDS. In addition, the mean lung injury score was significantly increased in malaria-infected mice with ALI/ARDS compared with that in malaria-infected mice without ALI/ARDS.

**Table 1:** Histopathological assessment of lung injury in malaria-infected mice without ALI/ARDS (n = 10) and malaria-infected mice with ALI/ARDS (n = 10).

Parameters	Score		*p* value
	Non-ALI/ARDS	ALI/ARDS	
Alveolar oedema	0.00 ± 0.00	1.90 ± 0.23	< 0.001*
Alveolar haemorrhage	1.20 ± 0.13	2.20 ± 0.13	< 0.001*
Septal thickening	1.20 ± 0.13	2.90 ± 0.10	< 0.001*
Leukocyte infiltrate	1.00 ± 0.00	2.80 ± 0.13	< 0.001*
Lung injury score	3.40 ± 0.16	9.80 ± 0.25	< 0.001*

The data are expressed as the mean ± SEM.

*Significant difference of *p* < 0.001 compared with non-ALI.

### Expression of SphK-1 and S1PR-3 in lung tissue of malaria-infected mice

**Fig 2:**
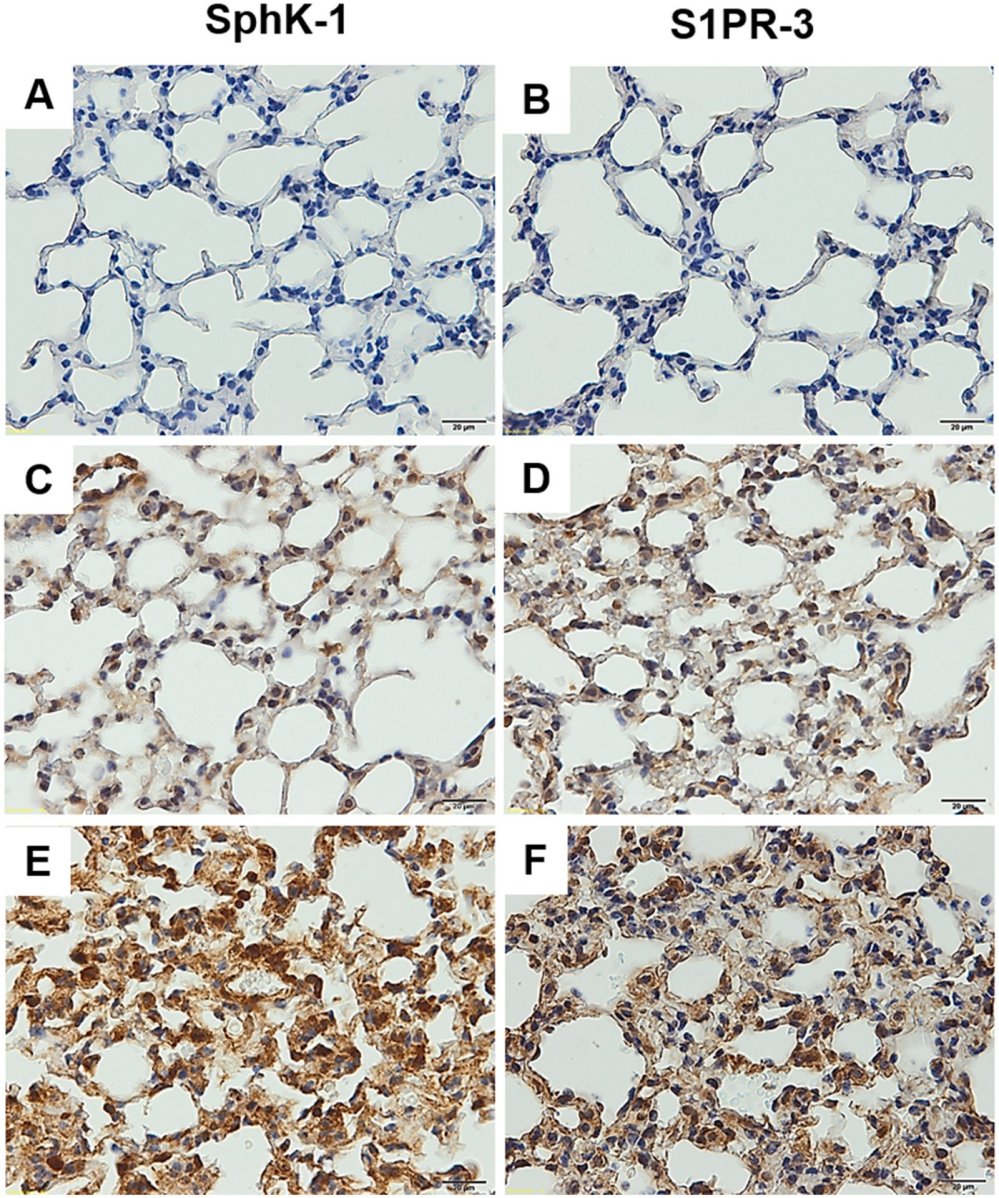
Immunohistochemistry staining for SphK-1 and S1PR-3 in the lung tissues of malaria-infected mice with ALI/ARDS demonstrated more intense cytoplasmic staining of SphK-1 and S1PR-3 in endothelial cells, alveolar epithelial cells and alveolar macrophages than those of control mice and malaria-infected mice without ALI/ARDS. (A), (B) Negative immunohistochemistry staining for SphK-1 and S1PR-3 in the control mice (n = 10). (C), (D) Weak expression of SphK-1 and S1PR-3 in the lung tissues of malaria-infected mice without ALI/ARDS (n = 10). (E), (F) The lung tissues from malaria-infected mice with ALI/ARDS (n = 10) demonstrated intense cytoplasmic staining of SphK-1 in luminal endothelial cells of vascular blood vessels and alveolar epithelial cells and in alveolar macrophages located in the alveolar space and septum (E). Intense cytoplasmic staining of S1PR-3 was evident in vascular endothelial cells, alveolar epithelial cells and alveolar macrophages (F).

**Fig 3:**
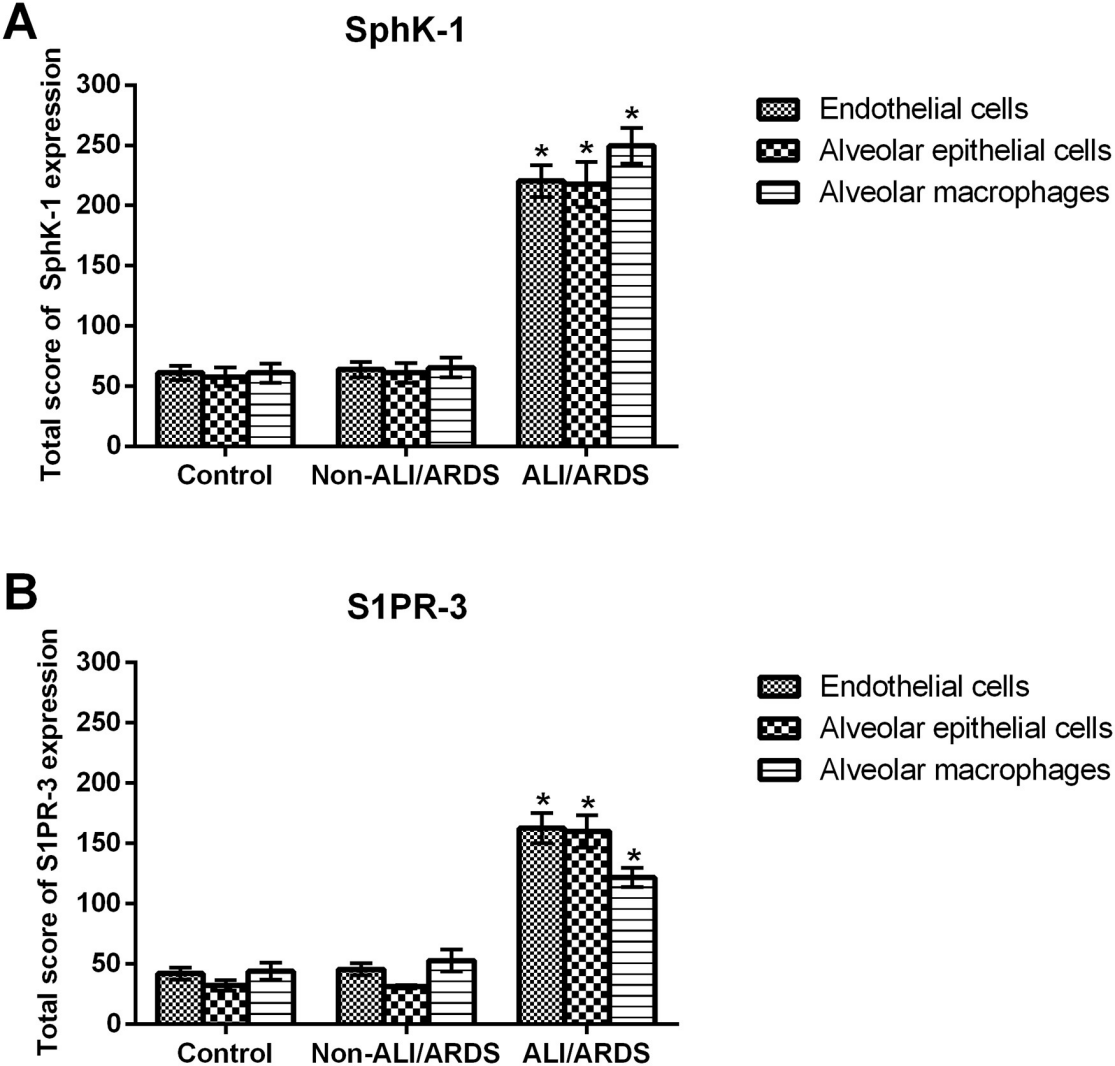
SphK-1 and S1PR-3 expression levels in endothelial cells, alveolar epithelial cells, and alveolar macrophages were significantly higher in the lung tissues of the ALI/ARDS group than in those of control mice and malaria-infected mice without ALI/ARDS. The mean total scores of (A) SphK-1 and (B) S1PR-3 expression in endothelial cells, alveolar epithelial cells, and alveolar macrophages were significantly higher in the lung tissues of the ALI/ARDS group **(n = 10)** compared with those of the non-ALI/ARDS **(n = 10)** and control **(n = 10)** groups. Note: *Significance of *p* < 0.001 compared with the control group and non-ALI/ARDS group. The data were analysed using the Mann-Whitney U test and are presented as the mean ± SEM.

For immunohistochemistry staining, the lung tissues from malaria-infected mice with ALI/ARDS demonstrated intense cytoplasmic staining of SphK-1 in luminal endothelial cells of vascular blood vessels and alveolar epithelial cells (Fig 2E). Furthermore, intense cytoplasmic staining was also observed in alveolar macrophages, which are located in the alveolar space and septum (Fig 2E). In addition, strong cytoplasmic staining of S1PR-3 was evident in vascular endothelial cells, alveolar epithelial cells and alveolar macrophages (Fig 2F). In comparison, only weak expression of SphK-1 and S1PR-3 was detected in the lungs of non-ALI/ARDS (Fig 2C and 2D) and control (Figs 2A and 1B) mice. For semi-quantitative analysis, the mean percentage of positively stained cells for SphK-1 was significantly increased in endothelial cells (80.19 ± 4.26%), alveolar epithelial cells (78.60 ± 2.51%) and alveolar macrophages (83.21 ± 4.94%) in the lung tissues of the ALI/ARDS group compared with that in the non-ALI/ARDS group (endothelial cells: 64.04 ± 6.38%; alveolar epithelial cells: 53.90 ± 3.52%, alveolar macrophages: 47.08 ± 2.24%) and the control group (endothelial cells: 61.22 ± 5.83%; alveolar epithelial cells: 57.99 ± 7.66%, alveolar macrophages: 61.00 ± 7.80%) (all *p* < 0.05). The mean percentage of positively stained cells for S1PR-3 was significantly increased in endothelial cells (64.15 ± 1.86%), alveolar epithelial cells (60.03 ± 2.72%) and alveolar macrophages (48.69 ± 4.89%) in the lung tissues of the ALI/ARDS group compared with that in the non-ALI/ARDS group (endothelial cells: 38.61 ± 2.09%; alveolar epithelial cells: 31.01 ± 1.45%, alveolar macrophages: 33.95 ± 3.57%) and the control group (endothelial cells: 35.674 ± 2.49%; alveolar epithelial cells: 27.07 ± 2.47%, alveolar macrophages: 27.05 ± 3.75%) (all *p* < 0.001). The mean total scores of SphK-1 and S1PR-3 expression in endothelial cells, alveolar epithelial cells, and alveolar macrophages are shown in Fig 3. The mean total score of SphK-1 and S1PR-3 expression was significantly upregulated in endothelial cells, alveolar epithelial cells, and alveolar macrophages in the lung tissues of the ALI/ARDS group compared with that in the non-ALI/ARDS and control groups (all *p* < 0.001).

**Fig 4:**
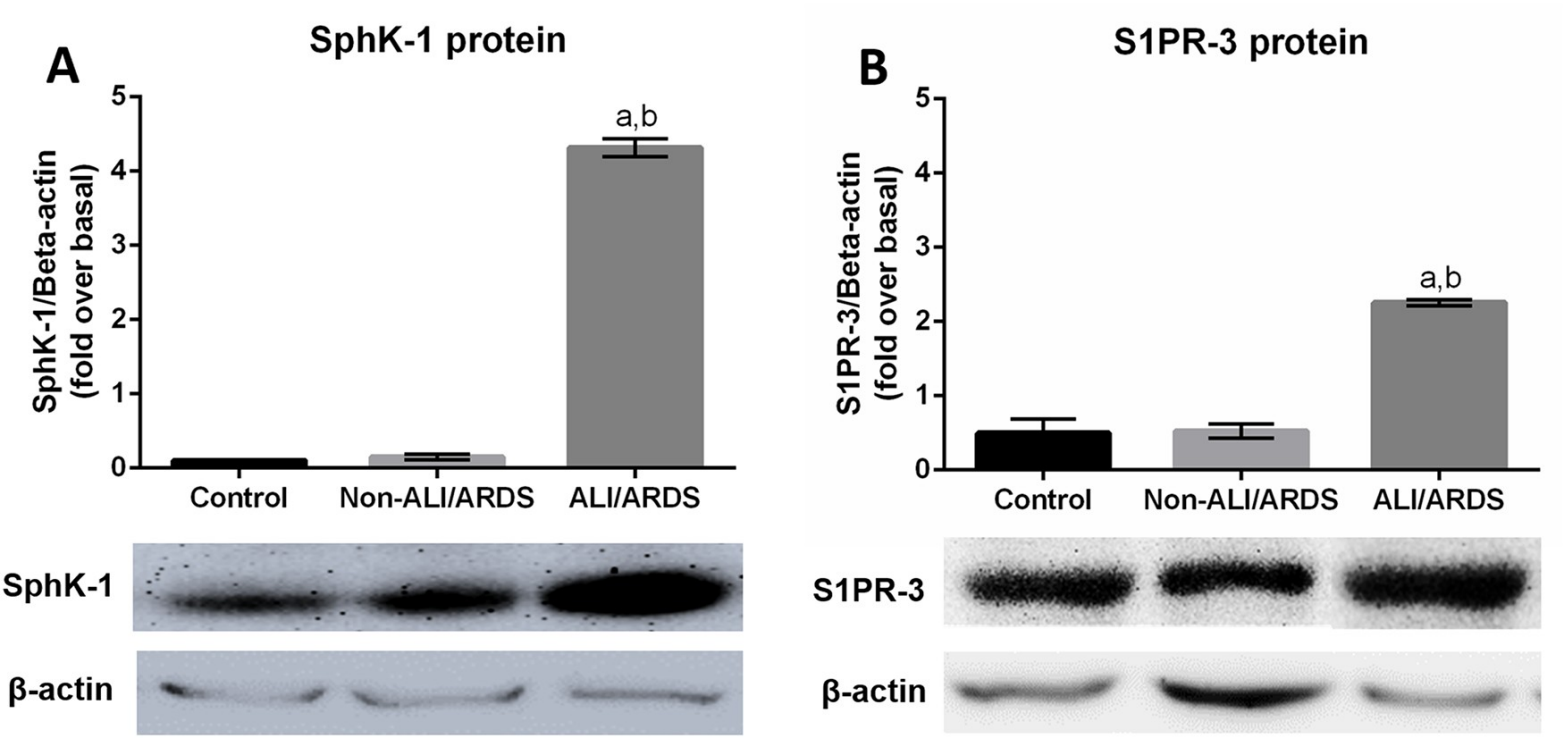
Western blot analysis confirmed that expression of the SphK-1 and S1PR-3 proteins was significantly increased n the lung tissue of malaria-infected mice with ALI/ARDS compared with that in the control mice and malaria-infected mice without ALI/ARDS. Representative immunoblots and graphs for SphK-1 (A) and S1PR-3 (B) are shown. The graphs represent the mean ± SEM of three independent experiments and are reported as protein expression normalized to beta (β) actin, expressed as fold change over basal level. Note: ^a^Significance of *p* < 0.001 compared with the control group. ^b^Significance of *p* < 0.001 compared with the non-ALI/ARDS group. The data were analysed using the Mann-Whitney U test.

The increased expression of S1P-3 and SphK-1 was confirmed at the protein level by using Western blot analysis (Fig 4). The molecular weight of the target proteins was 43 kDa for SphK-1 and 45 kDa for S1PR-3, which were detected in the lung tissue of the ALI/ARDS, non-ALI/ARDS and control groups. The expression levels of SphK-1 and S1PR3 in the lung tissues of the ALI/ARDS group were significantly higher than those of the non-ALI/ARDS and control groups (*p* < 0.05).

#### Levels of S1P in plasma and lung tissue

**Fig 5:**
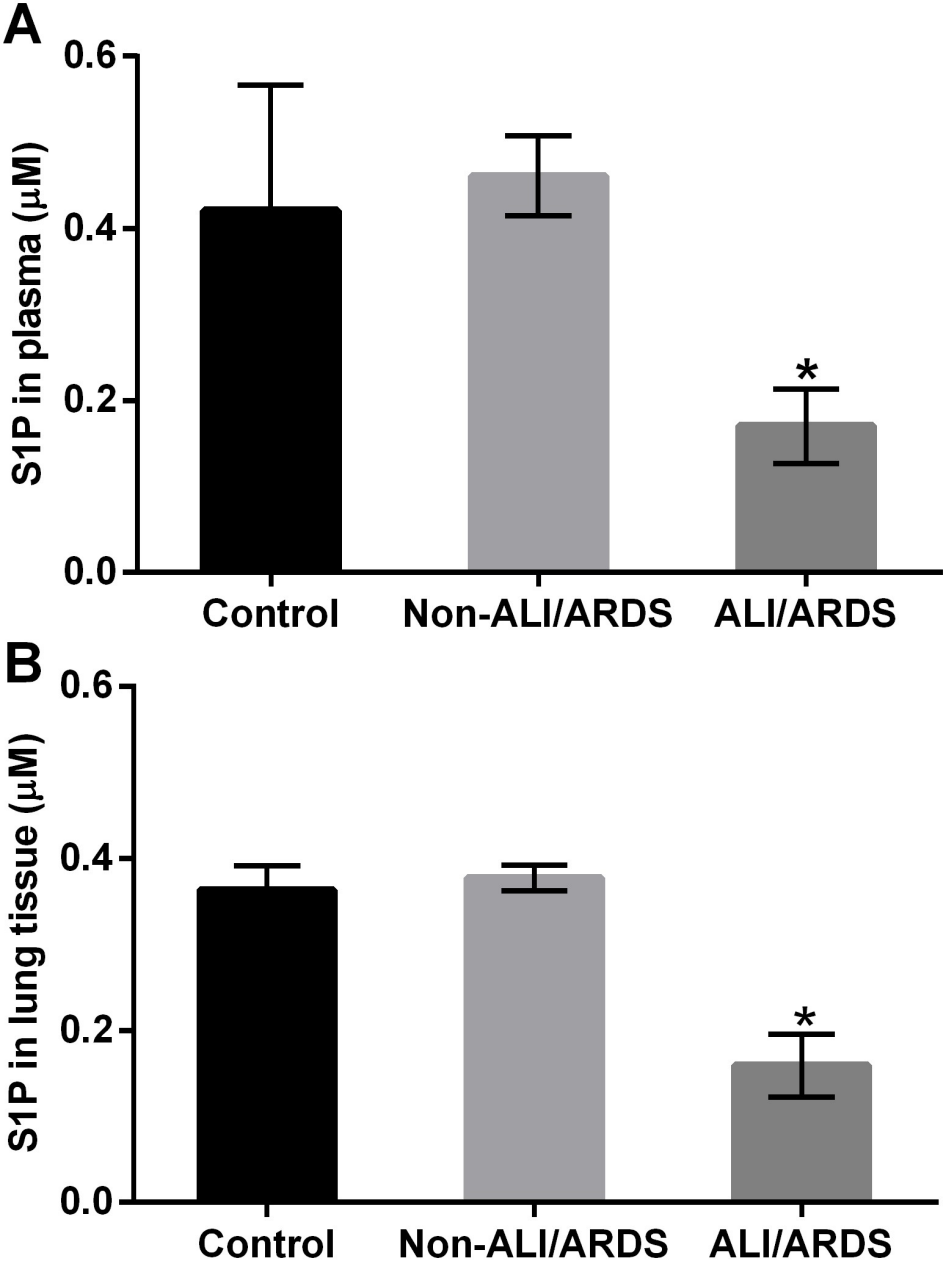
S1P concentration in plasma and lung tissue in malaria-infected mice with ALI/ARDS was significantly lower than that in the malaria-infected mice with non-ALI/ARDS and the control mice. (A) S1P levels in plasma and (B) S1P levels in the lung tissue. Note: *Significance of *p* < 0.001 compared with the non-ALI/ARDS and control group by the Mann-Whitney U test. The data are presented as the mean ± SEM.

The malaria-infected mice with ALI/ARDS showed a significant decrease in S1P in plasma and lung tissue compared with malaria-infected mice without ALI/ARDS and control mice. Likewise, we found that the S1P concentration in plasma and lung tissue in malaria-infected mice with ALI/ARDS was approximately twofold lower than that of the malaria-infected mice with non-ALI/ARDS and control mice (Fig 5).

### Correlation between SphK-1 and S1PR-3 in malaria-infected mice with ALI/ARDS

**Fig 6:**
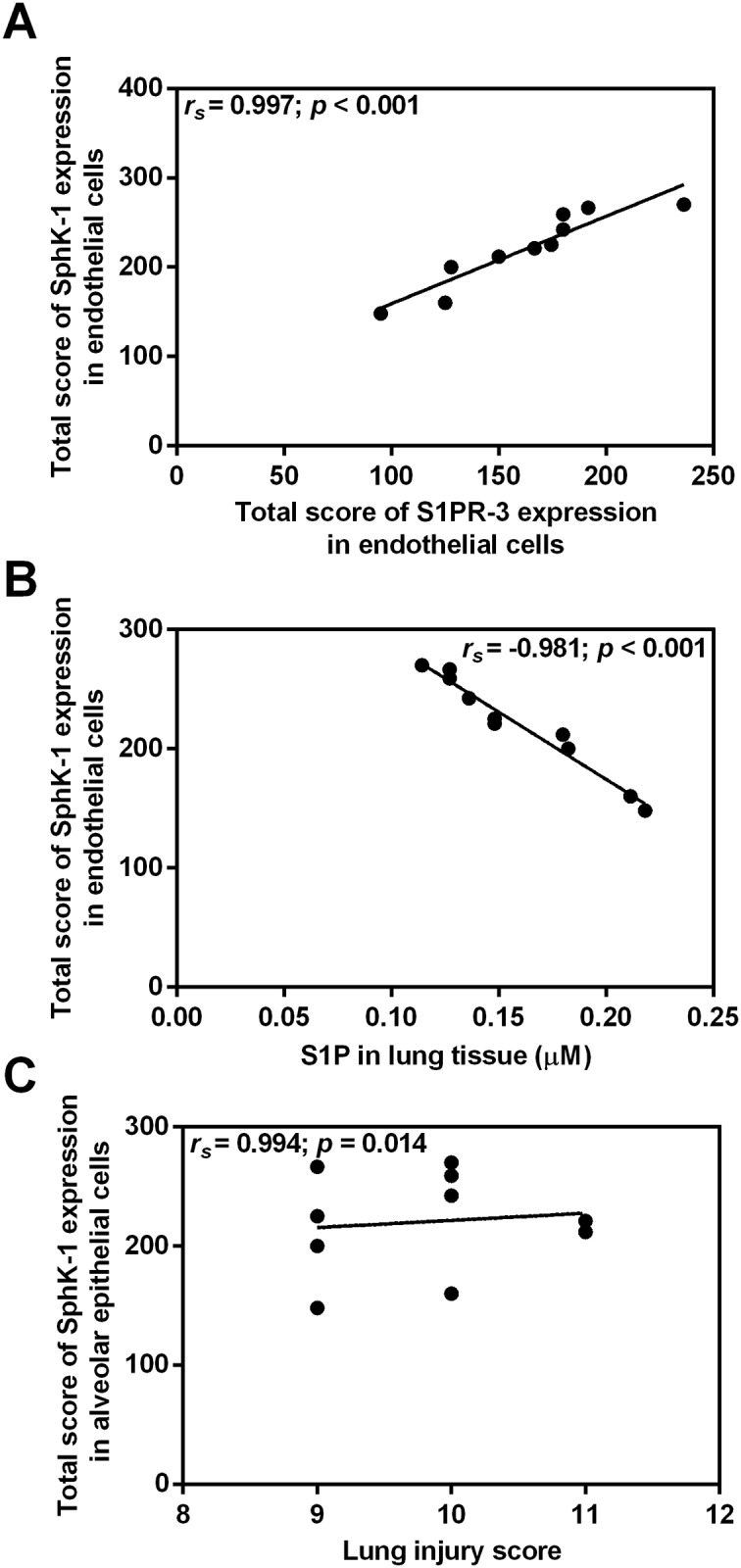
Positive correlations are demonstrated between SphK-1 expression and S1PR-3 expression in endothelial cells and between SphK-1 expression in alveolar epithelial cells and lung injury. **A negative correlation is demonstrated between SphK-1 expression in endothelial cells and S1P in lung tissue**. The total score for SphK-1 expression in endothelial cells was positively correlated with the total score for S1PR-3 expression in endothelial cells (A), the total score for SphK-1 expression in endothelial cells was negatively correlated with S1P in the lung tissue (B), and the total score for SphK-1 expression in alveolar epithelial cells was positively correlated with lung injury (C). Note: Correlation of SphK-1 expression in malaria-infected mice with ALI/ARDS (n = 10) using Spearman’s rank correlation test.

**Fig 7:**
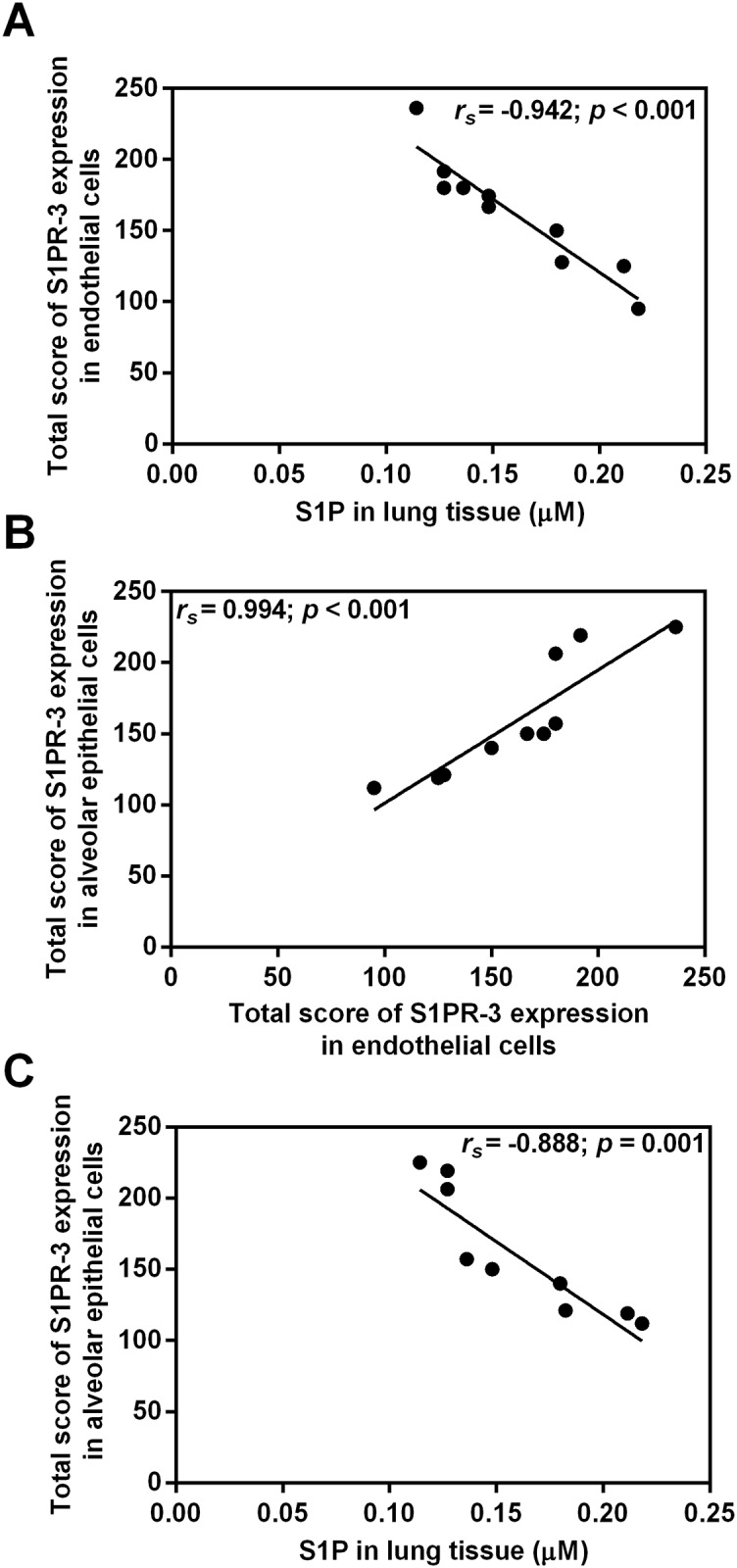
Negative correlations are demonstrated between S1PR3 in endothelial cells and S1P in lung tissue and between S1PR-3 in alveolar epithelial cells and S1P in lung tissue. **A positive correlation is demonstrated between S1PR-3 in alveolar cells and S1PR-3 in endothelial cells**. The total score for S1PR-3 expression in endothelial cells was negatively correlated with S1P in the lung tissue (A), the total score for S1PR-3 expression in alveolar cells was negatively correlated with the total score for S1PR-3 expression in endothelial cells (B), and the total score for S1PR-3 expression in alveolar epithelial cells was negatively correlated with S1P in the lung tissue (C). Note: Correlation of S1PR-3 expression in malaria-infected mice with ALI/ARDS (n = 10) using Spearman’s rank correlation test.

In malaria-infected mice with ALI/ARDS (n = 10), there was a positive correlation between the total score of SphK-1 and S1PR-3 expression levels in endothelial cells (Spearman’s rank correlation, *r*_*s*_ = 0.997; *p* < 0.001) (Fig 6A). The total score of SphK-1 expression in endothelial negatively correlated with S1P level in the lung tissues (Spearman’s rank correlation, *r*_*s*_ = -0.981; *p* < 0.001) (Fig 6B). In addition, a positive correlation was obtained between the total score of SphK-1 expression in alveolar epithelial cells and lung injury score (Spearman’s rank correlation, *r*_*s*_ = 0.994; *p* = 0.014) (Fig 6C). Regarding S1PR-3 expression in mouse lung tissues with malaria-associated ALI/ARDS, there were a significant negative correlation between S1P levels in lung tissues and the total score of S1PR-3 expression in endothelial cells (Spearman’s rank correlation, *r*_*s*_ = -0.942; *p* < 0.001) (Fig 7A) and in alveolar epithelial cells (Spearman’s rank correlation, *r*_*s*_ = -0.888; *p* = 0.001) (Fig 7C). In addition, the total score of S1PR-3 expression in alveolar epithelial cells significantly positively correlated with the total score of S1PR-3 expression in endothelial cells (Spearman’s rank correlation, *r*_*s*_ = 0.994; *p* < 0.001) (Fig 7B).

## Discussion

Our study provides the first extensive analysis of the expression and distribution of SphK-1 and S1PR-3 at the protein level in *P*. *berghei* ANKA-infected DBA/2 mice with ALI/ARDS. The present study showed enhanced SphK-1 expression in endothelial cells, alveolar epithelial cells and alveolar macrophages in the lung tissues of malaria-infected mice with ALI/ARDS. These results are consistent with evidence from previous studies, which reported immunopositive staining of SphK-1 at the apical surface of alveolar epithelial cells, foamy macrophages and endothelial cells lining the blood vessels in normal human lung tissue [28] and in a pulmonary endothelial cell model of acute pulmonary endothelial cell injury [29]. A previous study in mouse tissue reported that the highest activity of sphingosine kinase was found in the lung and thymus, whereas the lowest activity was expressed in skeletal muscle [17]. Studies suggest that SphK-1 plays a role in the regulation of lipopolysaccharide (LPS)-induced lung injury in *in vivo* experiments. In contrast to the protective role of SphKs in the endothelium [30], SphKs can promote a proinflammatory response in epithelial and immune cells [31]. In epithelial cells, activation of SphK-1 by tumour necrosis factor (TNF), interleukin-1 (IL-1), or LPS mediates the enhanced expression of cyclooxygenase-2 (COX-2) and monocyte chemoattractant protein-1 (MCP-1) [32]. Studies suggest that SphKs can promote inflammation or protect against injury in the lung, pointing to the different roles of SphKs and S1P in lung function. Furthermore, in human lung endothelial cells, overexpression of SphK-1, compared with SphK-2, significantly enhanced the conversion of sphingosine to S1P [30], suggesting a major role for SphK-1 in S1P formation. In addition, there were positive correlations between SphK-1 and S1PR-3 expression in endothelial cells and a positive relationship between SphK-1 expression in alveolar cells and lung injury. However, further investigations regarding the precise mechanism of increased expression of SphK-1 in malaria infection are needed. There could be several possible explanations for the alteration of SphK-1. This finding may be due to the changes in expression of SphK-2 and S1P lyase, which were not determined in this study. In addition, the synthesis of S1P is regulated by an availability of sphingosine, as the substrate. The measurement of sphingosine and ceramide levels should be determined in the lung tissue and plasma samples to explain the functional role of SphK-1/S1P /S1PR-3 pathway involved in the pathogenesis of malaria.

In contrast, this study demonstrated that S1P was significantly reduced in the plasma and lung tissue of malaria-infected mice with ALI/ARDS. This finding is consistent with previous studies showing decreased S1P levels in blood samples of malaria patients infected with *P*. *falciparum* [23, 24] and experimental malaria [23]. Our findings suggested that the reduction of S1P levels might be associated with the location of S1P synthesis, the duration of SphK-1 activation, which plays a role in the regulation of lung inflammation, and the reduction in the source of S1P, such as red blood cells and platelets, in severe malaria [24]. Normally, S1P can be dephosphorylated back to sphingosine by sphingosine phosphatase 1 and 2 (SGPP 1 and 2), or it can be irreversibly cleaved by S1P lyase [33]. In this study, we found negative correlations between S1PR-3 expression in alveolar epithelial cells and the level of S1P in lung tissues. Therefore, we hypothesized that the decreased level of S1P in malaria-infected mice with ALI/ARDS might be due to the concentration of S1P carrier proteins such as the low-density lipoprotein (LDL), high-density lipoprotein (HDL), and albumin fractions [34]. A systematic review and meta-analysis focused on serum lipids and lipoproteins in malaria reported that levels of HDL, LDL and lipoproteins are significantly lower in malaria patients compared to healthy controls, suggesting the loss of carrier proteins for S1P [35]. A previous study investigating the pulmonary endothelial cell model of acute injury induced by LPS demonstrated that both mesenchymal stem cells and S1P can alleviate ALI via regulation of S1P receptors and SphK, suggesting that S1P acts as a novel therapeutic strategy to improve clinical outcome in severe malaria [29]. In mice receiving intravenous S1P (1 μM final concentration) 1 h after LPS administration, the inflammatory cell infiltrate is markedly reduced. This effect demonstrates the potent ability of S1P to decrease vascular permeability to inflammatory cells *in vivo* [36].

In addition, strong immunoreactivity of S1PR-3 expression was evident in endothelial cells, alveolar epithelial cells and alveolar macrophages in the lung tissues of malaria-infected mice with ALI/ARDS. Increased S1PR-3 expression as observed using immunohistochemistry staining was consistent with the Western blot results. S1P has a diverse range of functions, which are mediated in a receptor-dependent manner through G-protein coupled receptors [33]. S1PR-3 is an S1P receptor predominantly found in many tissues, including the lung [37], liver [38] and heart [39], and a crucial regulator of vascular permeability [20, 40]. A previous study in models of ARDS showed that S1PR-3 promotes pulmonary inflammation in the late phase of sepsis via PAR1-S1PR-3 cross talk, propagating the dissemination of IL-1 and tissue factor to the lungs [41]. S1P, acting on the S1PR-3 receptor expressed on both type I and type II alveolar epithelial cells but not on the vascular endothelium, induces pulmonary oedema by acute tight junction opening and zonula occludens-1 (ZO-1) loss, likely through Rho activation [37]. In addition, the plasma S1PR-3 protein level has been proposed as a biomarker of severity in critically ill ARDS patients [21, 40]. Therefore, our findings confirmed that high S1PR-3 expression is associated with the development of malaria-associated ALI/ARDS in mice.

The present study suffered from the following limitations. This study did not determine the level of S1PR-3 in plasma; therefore, we could not demonstrate the time course of S1PR-3 expression in malaria-infected mice with ALI/ARDS. Further studies should focus on the kinetics of S1P, SphK-1 and S1PR-3 expression *in vivo* and *in vitro*. Moreover, further studies of S1PR-3 antagonists/blockers in malaria-infected mice with ALI/ARDS would help to confirm the involvement of S1PR-3 and SphK-1 in malaria-infected mice with ALI/ARDS. In addition, to demonstrate that SphK-1 and S1PR-3 are indeed involved in the development of ALI/ARDS, further work should focus on the same infection model performed in SphK-1- and S1PR-3-knockout mice. Finally, to definitely demonstrate how SphK-1 and S1PR-3 are involved in malaria-associated ALI/ARDS, global gene expression analysis should be performed to identify the signalling pathways involved with SphK-1 and S1PR-3.

## Conclusion

This study provides new insights into the expression of SphK-1 and S1PR-3 in *P*. *berghei* ANKA-infected DBA/2 mice with ALI/ARDS. The concentration of S1P in plasma and lung tissues was significantly decreased in malaria-infected mice with ALI/ARDS. Increased expression of the SphK-1 and S1PR-3 proteins significantly correlated with the lung injury score and S1P concentration. These results suggest that the increased expression of SphK-1 and S1PR-3 in lung tissues is implicated in development of ALI/ARDS during malaria infection.
